# Secernin-1 Contributes to Colon Cancer Progression through Enhancing Matrix Metalloproteinase-2/9 Exocytosis

**DOI:** 10.1155/2015/230703

**Published:** 2015-02-26

**Authors:** Shengtao Lin, Tao Jiang, Yang Yu, Huamei Tang, Su Lu, Zhihai Peng, Junwei Fan

**Affiliations:** ^1^Department of General Surgery, Shanghai First People's Hospital, School of Medicine, Shanghai Jiao Tong University, Shanghai 200080, China; ^2^Department of Anal-Colorectal Surgery, General Hospital of Ningxia Medical University, Yinchuan 750004, China; ^3^Department of Pathology, Shanghai First People's Hospital, School of Medicine, Shanghai Jiao Tong University, Shanghai 200080, China

## Abstract

Emerging evidence shows that exocytosis plays a key role in tumor development and metastasis. Secernin-1 (SCRN1) is a novel regulator of exocytosis. Our previous work identified SCRN1 as a tumor-associated gene by bioinformatics analysis of transcriptomes. In this study, we demonstrated the aberrant overexpression of SCRN1 at mRNA and protein level in colon cancer. We also revealed that overexpression of SCRN1 was significantly associated with the tumor development and poor prognosis. Experiments *in vitro* validated that SCRN1 may promote cancer cell proliferation and secretion of matrix metalloproteinase-2/9 (*MMP*-2/9) proteins to accelerate tumor progression.

## 1. Introduction

Colon cancer is one of the most important causes of cancer morbidity and mortality globally, and the incidence is increasing in the Asia-Pacific region [1, 2]. In China, the colon cancer incidence rate is increasing due to changes in individual lifestyle, nutritional habits, and environment [3]. Currently, surgical resection, chemotherapy, radiotherapy, and other curative strategies are applied to cure colon cancer. In spite of the advances in screening, diagnosis, and treatment, some patients with colon cancer have poor prognosis due to lymph node metastasis (LNM) and distant metastasis [4, 5]. Therefore, it is of great significance to further investigate the molecular mechanism of occurrence, development, and metastasis in colon cancer. Molecular genetics studies have revealed some critical tumor-associated genes underlying the progression of colon cancer [6]. In a previous study, many differentially expressed genes were identified and some serve as biomarkers in colon cancer [7]. Nevertheless, the roles of these novel biomarkers in colon cancer progression remain poorly understood.

In a previous study, a novel cytosolic protein, Secernin-1 (SCRN1), was identified as a regulator of exocytosis in mast cells [8]. SCRN1 was identified as a prognostic biomarker for synovial sarcoma and could accurately predict the overall and metastasis-free survival rates of patients [9]. Moreover, SCRN1 was shown to be overexpressed in gastric cancer cell lines and showed potential as a novel immunotherapy target [10]. Recent research showed that SCRN1 mRNA is highly expressed in colon cancerous regions and that high expression of SCRN1 mRNA resulted in poor prognosis [11]. However, the expression pattern and cellular localization of SCRN1 protein, its clinical significance, and its mechanism of action in the progression of colon cancer remain poorly understood.

Exocytosis is a process by which cells release material within membrane-limited vesicles by fusion of the vesicles with the cell membrane. The molecular mechanisms underlying the metastasis of colon cancer are heterogeneous [5]. Emerging evidence indicates that exocytosis participates in tumor growth, migration, and metastasis [12–14]. Secretory products from both cancer cells and host cells form parts of a signaling network that initiates invasive tumor growth [15]. Matrix metalloproteinases (*MMP*s) are zinc-dependent endopeptidases that can degrade various extracellular matrix components. Several studies demonstrated that regulation of* MMP*s secretion leads to the acceleration of tumor invasion [16–19].* MMP*-2 and* MMP*-9 promote the degradation of the extracellular matrix, proliferation [20–22], and invasion of colon cancer cells [23–26]. We hypothesized that, in colon cancer cells, SCRN1 regulates the secretion of* MMP*s to promote cell proliferation and invasion.

In the present study, we investigated SCRN1 gene expression at protein and mRNA level in 40 colon cancer tissues paired with adjacent normal mucosa. SCRN1 protein expression in tissue microarrays (TMAs) was also detected to assess the expression pattern in colon cancer tissue. Furthermore, we analyzed the relationship between SCRN1 expression and clinicopathological features and investigated whether SCRN1 could be a predictor of prognosis for patients with colon cancer. Experiments in colon cancer cell lines were carried out to study SCRN1 biological functions* in vitro*. Differences in* MMP*-2/9 mRNA expression and* MMP*-2/9 protein secretion were measured to evaluate the effect of SCRN1 on* MMP*-2/9 expression and secretion.

## 2. Materials and Methods

### 2.1. Patients and Specimens

The study was approved by the Ethics Committee of Central Hospital of Shanghai Hongkou District. Fifty-five male and sixty-two female colon cancer patients who had undergone surgical resection were enrolled in this study. Forty fresh tissues together with 117 formalin-fixed paraffin-embedded tissues (FFPE) from Central Hospital of Shanghai Hongkou District were used in this study, and informed consent was obtained from all patients. These fresh specimens were subpackaged, immediately frozen in liquid nitrogen, and subsequently stored at −80°C. All patients underwent surgical resection between January 2002 and December 2007. All specimens were analyzed and a diagnosis of colon cancer was made by at least two pathologists. All cancer specimens were graded in accordance with the World Health Organization criteria, and tumor staging was conducted as indicated by the American Joint Committee on Cancer's (AJCC) seventh edition cancer staging system. The patient follow-up after surgery was conducted under the guidelines of the National Comprehensive Cancer Network Practice. Disease-free survival (DFS) and overall survival (OS) rates were defined as the interval from the initial surgery to clinically or radiologically proven recurrence, metastasis, or death. The final follow-up was conducted in June 2013.

### 2.2. RNA Extraction and Quantitative Real-Time Polymerase Chain Reaction (qPCR)

Total RNA from 40 frozen colon cancer tissues paired with adjacent normal mucosa was extracted according to the manufacturer's protocol (RNAEasy Kit, Qiagen, Hilden, Germany), and single-stranded cDNAs were synthesized according to the provider's instruction (High Capacity cDNA Reverse Transcription Kit, Applied Biosystems, Carlsbad, CA, USA). qPCR was performed with SYBR Green PCR Master Mix (Applied Biosystems) and Mastercycler ep realplex (Eppendorf, Hamburg, Germany). The SCRN1 gene was amplified using the sense primer 5′-GGATGGTCTGGTGGTATTTGG-3′ and antisense primer 5′-CCTTGGAACTTGGTCGATTG-3′. The human glyceraldehydes-3-phosphate dehydrogenase (GAPDH) gene was amplified as an endogenous control using the sense primer 5′-AGCAAGAGCACAAGAGGAAG-3′ and the antisense primer 5′-AACTGGTTGAGCACAGGGTA-3′. These reactions were repeated three times. The fold change (2^−ΔΔCt^) of SCRN1 expression was calculated using the following formulas: SCRN1ΔCt = (mean SCRN1_Ct − mean GAPDH_Ct), SCRN1ΔΔCt = (SCRN1ΔCt_tumor − SCRN1ΔCt_nontumor) for each specimen.

Similar procedures were conducted to measure the effect of SCRN1 on* MMP*-2/9 mRNA expression in colon cell lines. The primers used in the qPCR were as follows:* MMP*-2 sense, 5′-GATGCCGCCTTTAACTGG-3′ and antisense 5′-TCAGCAGCCTAGCCAGTCG-3′;* MMP*-9 sense, 5′-TCTGGAGGTTCGACGTGAAG-3′ and antisense 5′-GGGCACTGCAGGATGTCATA-3′. The GAPDH primers used are the same as mentioned above. These reactions were repeated three times. The fold change (2^−ΔCt^) of* MMP-*2 and* MMP-*9 mRNA expression was calculated for each sample.

### 2.3. Western Blot Analysis

Total protein was extracted from fresh-frozen colon cancer tissues and paired adjacent normal colon tissues using RIPA lysis buffer and the concentration was measured with BCA protein assay kit (Beyotime Biotechnology, Jiangsu, China) according to the manufacturer's instructions. Equivalent amounts of protein (35 *μ*g) were electrophoresed on a 12% sodium dodecyl sulfate-polyacrylamide gel for 1.5 h and then transferred to polyvinylidene difluoride membranes (Santa Cruz, Biotechnology, USA) according the standard protocols. The membrane was blocked with 5% nonfat milk at room temperature for 1 h followed by incubating at 4°C overnight with appropriate primary antibodies: SCRN1 (1 : 100 dilution, Anti-SCRN1 antibody ab104055, Abcam, Cambridge, MA, USA) and GAPDH (1 : 2000 dilution, Beyotime Biotechnology, Jiangsu, China). After washing with TBST, the membrane was incubated with secondary antibody-horseradish peroxidase conjugate (1 : 2000 dilution, Beyotime Biotechnology, Jiangsu, China). The bands were visualized using ECL chemiluminescence kit (Applygen Technologies Inc., Beijing, China). The GAPDH expression was used to normalize equal loading of the samples. Similar procedures were performed when assessing the inhibition efficiency of SCRN1 silence in RKO and HCT116 cells.

### 2.4. Tissue Microarray (TMA) Construction

Colon cancer tissue sections, stained with hematoxylin and eosin, were screened for appropriate tumor tissue, adjacent tissue (at least 2 cm from the tumor), and related lymph nodes. The TMAs were constructed in cooperation with Outdo Biotech Co. (Shanghai, China). Two cores were archived from each formalin-fixed, paraffin-embedded specimen and from each adjacent tissue specimen with the help of a 2.0 mm diameter punch instrument. Additionally, at least one core from lymph node metastasis (LNM) was similarly archived. In order to assure that all specimens from the same patient were treated uniformly, the samples derived from the same patient were placed next to each other on the TMA.

### 2.5. Immunohistochemical Analysis

Immunohistochemistry was conducted on TMA sections (4 *μ*m) using Envision kit (Dako, Glostrup, Denmark). The antigen retrieval was carried out in preheated citrate buffer for 30 min. Specimens were incubated with a primary antibody against SCRN1 (Anti-SCRN1 antibody ab104055, Abcam, Cambridge, MA, USA; diluted 1 : 100) overnight at 4°C. The slides were then incubated with a goat-anti-rabbit secondary antibody for 30 min at room temperature. Immunoreactivity was evaluated by two pathologists blinded to the patient clinical information. Positive staining was classified into four groups: negative, weakly positive, moderately positive, and strongly positive on the basis of the staining intensity and extent. Strongly positive and moderately positive specimens were regarded as high SCRN1 expression specimens, while weakly positive and negative specimens were regarded as low SCRN1 expression specimens.

### 2.6. Cell Culture and Small Interfering RNA (siRNA) Transfection

The human colon cancer cell lines, RKO and HCT116, were cultured in Dulbecco's modified Eagle medium (DMEM, Gibco, Carlsbad, CA, USA) with 10% fetal bovine serum (FBS, Gibco), 1% streptomycin, and penicillin, at 37°C under a 5% humidified CO_2_ atmosphere. Double-stranded RNA duplexes targeting human SCRN1 and a scrambled siRNA used as a negative control were synthesized. Transfections were performed using Lipofectamine 2000 (Invitrogen, Carlsbad, CA, USA) following the manufacturer's instructions. Briefly, 1 × 10^5^ cells were seeded in each well of a 6-well plate. Once the cells reached 80% confluence, the transfection was performed with siRNA using the transfection reagent in serum-free medium. After 48 h, the cells were harvested for further analysis. The inhibition efficiency was confirmed by western blot.

### 2.7. Cell Proliferation Assay and Plate Clone Formation Assay

The 3-(4,5-dimethylthiazol-2-yl)-2,5-diphenyltetrazolium bromide (MTT) reagent was applied to assess the cell proliferation ability. Cells were harvested at the logarithmic phase and seeded into 96-well plates (2000 cells/well) for MTT assay. The cells were incubated for 5 days. Every well was added 200 *μ*L dimethyl sulfoxide (DMSO) to dissolve the formazan after being cultured with MTT solution for 2 h. The absorbance was read at 490 nm to measure the cell quantity.

When performing the clone formation assay, exponentially growing cells were collected and seeded in 6-well plates at a density of 1000 cells per well. After 14 days of incubation, the colonies were stained with Giemsa for 20 min. Colonies were counted, and the plates were photographed.

### 2.8. Transwell Invasion Assay

Cell invasion assays were performed using transwell chambers. Cells were cultured in serum-free DMEM for 12 h before being trypsinized and seeded into the upper chamber containing a polycarbonate membrane coated with Matrigel. DMEM with 10% FBS, which was added to the lower chambers, was used as a source of chemoattractant. The noninvading cells in the upper chamber were removed with cotton swabs after incubation at 37°C with 5% CO_2_ for 48 h. Cells on the lower surface were stained with Giemsa after being fixed with 5% formalin. Photographs were captured in 3 different fields and the quantity of cells that invaded through the membrane was counted from 3 randomly selected fields. All experiments were repeated 3 times.

### 2.9. Enzyme-Linked Immunosorbent Assay (ELISA)

Cells were cultured and the supernatant was collected. ELISA was performed using ELISA detection kits (Life Technology Co., Grand Island, NY, USA) for* MMP*-2 and* MMP*-9 detection according to the manufacturer's guidelines. The sample concentration was determined based on the regression equation established using serial dilutions of standards.

### 2.10. Statistical Analysis

All statistical analyses were performed using the SPSS statistical software program version 19.0 (SPSS, Chicago, IL, USA). The *χ* ^2^ test or Fisher's exact test was applied to estimate the relationship between SCRN1 expression and specimen clinicopathological features. Kaplan-Meier curves with log-rank tests were used to present the cumulative survival proportion for DFS and OS according to SCRN1 expression level. Furthermore, univariate and multivariate Cox proportional hazard regressions were carried out to estimate the hazard ratios for the study variables. Differences between the groups were analyzed by *t*-test. A *P* value of less than 0.05 was regarded to be of statistical significance and a *P* value less than 0.01 was regarded to be apparently statistically significant.

## 3. Results

### 3.1. SCRN1 Was Upregulated in Colon Cancer

**Figure 1:**
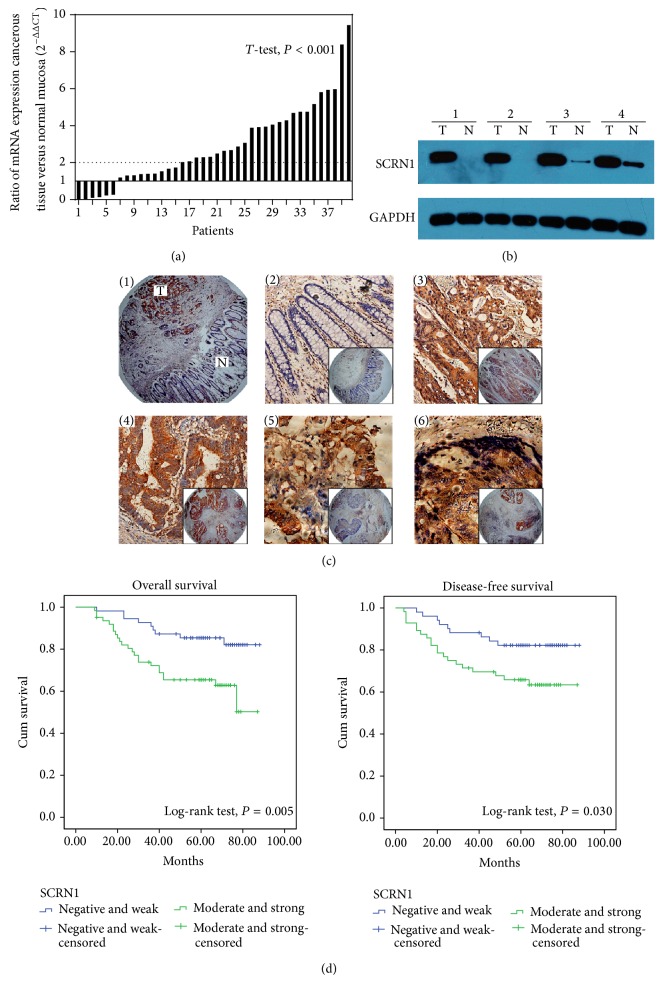
SCRN1 expression in colon cancer and Kaplan-Meier survival curves of OS and DFS. (a) SCRN1 mRNA expression analysis using qPCR in 40 paired colon cancerous tissues and adjacent normal mucosa. For each sample, the relative SCRN1 mRNA level was normalized using GAPDH expression. (b) Western blot analysis was performed to examine SCRN1 protein expression in 4 representative cases of primary colon cancer and paired normal tissue. (c) Immunohistochemistry revealed SCRN1 expression on tissue microarray (negative SCRN1 expression in normal colonic epithelium, weak expression in well differentiated tumor, moderate and strong expression in poorly differentiated colon cancer, and strong expression in lymph node metastasis.) (d) Kaplan-Meier survival curves of OS and DFS according to SCRN1 expression.

SCRN1* mRNA* expression was confirmed by qPCR in 40 colon cancerous tissues and adjacent normal mucosa. Among the 40 specimens of colon cancer, 34 showed higher SCRN1 expression compared with paired normal mucosa. In addition, 25 specimens presented more than 2-fold upregulation of SCRN1 mRNA (Figure 1(a)). The relative expression (ΔCt) of SCRN1 mRNA was 3.68 ± 3.49 in cancerous tissue and was 4.34 ± 3.15 in normal mucosa. Western blot revealed that SCRN1 protein expression was elevated in colon cancer tissue compared with adjacent normal mucosa (Figure 1(b)). The result was in accordance with our previous work using bioinformatics analysis [7] and further confirmed SCRN1 upregulation in colon cancer [11].

### 3.2. Correlation between SCRN1 Expression and Clinical Pathological Features in Colon Cancer

SCRN1 brown staining was mainly observed in the cytoplasm of colon epithelial, mesenchymal, and cancer cells (Figure 1(c)). SCRN1 protein expression was significantly different between normal mucosa and cancerous tissue (*P* < 0.001, Table 1) and LNM tissue (*P* < 0.001, Table 1). Out of 117 cancerous tissues, 62 tissues (53.0%) showed moderate and strong SCRN1 expression, which contrasted with the low SCRN1 expression observed in normal mucosa tissue. Only 34 normal mucosa tissues (29.1%) showed moderate and strong SCRN1 expression. Moreover, 25 out of 42 LNM tissues (59.5%) showed moderate and strong SCRN1 expression. SCRN1 protein expression was higher in cancerous tissues and LNM tissues than in normal mucosa. The relationship between clinicopathological features and SCRN1 expression is presented in Table 2 (117 patients). No significant correlation was found between SCRN1 expression and age, gender, location, differentiation, and vessel invasion. SCRN1 expression was correlated with T stage (*P* = 0.013), N stage (*P* = 0.023), distant metastasis (*P* = 0.025), and AJCC stage (*P* = 0.018). These results suggested that SCRN1 might be a key regulatory factor in colon cancer progression. Taken the expression pattern and clinical pathological significance into consideration, we hypothesized that SCRN1 contributes to the colon cancer progression through accelerating cancer cell proliferation and invasion.

### 3.3. Survival Analysis and Prognostic Significance of SCRN1 Expression

Kaplan-Meier survival curves with the log-rank test for OS and DFS were undertaken to elucidate the relationship between colon cancer SCRN1 expression and patient survival (Figure 1(d)). The estimated mean OS and DFS were significantly different between patients with differential SCRN1 expression. The estimated mean OS time was 79.10 ± 2.81 months for patients with negative and weak SCRN1 expression and was 63.69 ± 3.91 months for patients with moderate and strong SCRN1 expression (*P* = 0.005). Similar results were observed in the estimated mean DFS time (77.32 ± 3.32 months compared with 63.72 ± 4.34 months, *P* = 0.030). OS and DFS rates decreased with increasing SCRN1 expression.

In univariate analysis, T stage, N stage, distant metastasis, AJCC stage, differentiation, vessel invasion, and SCRN1 expression were associated with OS (Table 3). Similarly, N stage, distant metastasis, AJCC stage, differentiation, and SCRN1 expression were associated with DFS (Table 4). To further investigate the relationship between patient prognosis and individual parameters, multivariate analysis was performed using the Cox proportional hazards model for all significant factors in the univariate analysis. We excluded T stage, N stage, and distant metastasis from the final model because these factors were collinear with AJCC stage. The results showed that SCRN1 expression (*P* = 0.015), differentiation (*P* < 0.001), and AJCC stage (*P* < 0.001) were confirmed as independent prognostic factors for OS (Table 3). In addition, SCRN1 expression (*P* < 0.048), differentiation (*P* < 0.001), and AJCC stage (*P* < 0.001) were also verified as independent prognostic factors for DFS (Table 4).

### 3.4. Silencing of SCRN1 Expression Inhibited Cell Proliferation and Reduced Efficiency of Clone Formation* In Vitro*

**Figure 2:**
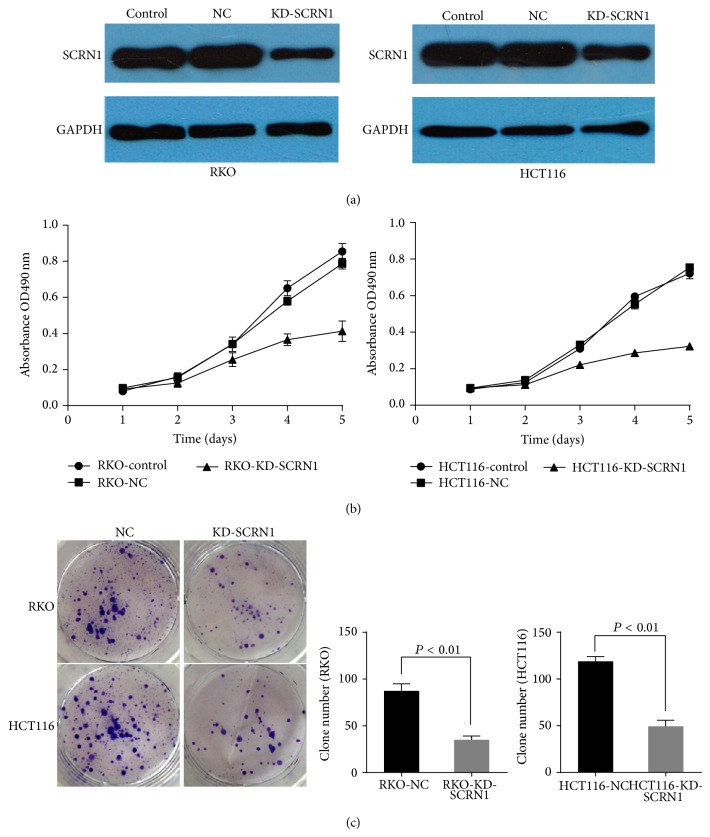
Silencing SCRN1 expression in colon cancer cell inhibited cell proliferation, clone formation. (a) Western blot analysis validated the inhibition efficiency of SCRN1 knockdown in RKO and HCT116 cells. (b) MTT assay revealed the inhibition of proliferation by SCRN1 silence in both RKO and HCT116 cells. Data was presented as mean ± SD. (c) Clone formation assay revealed the inhibition of clone formation ability by SCRN1 silence in both RKO and HCT116 cells. Data was presented as mean ± SD, *P* < 0.01 in both cases.

SCRN1 expression was knocked down by siRNA in human colon cancer cells RKO and HCT116 (Figure 2(a)). The cells transiently transfected with SCRN1 siRNA were used to perform proliferation assays. The MTT proliferation assay revealed cell growth inhibition in KD-SCRN1 cells (Figure 2(b)). Clone formation assays were performed to investigate the role of SCRN1 in clone formation. Results indicated that the ability to form colonies was reduced in KD-SCRN1 cells compared with NC cells (*P* < 0.01 in both cases) (Figure 2(c)). However, the mechanism of its effect on cell proliferation and clone formation remains unclear. SCRN1 promotes exocytosis in cells and multiple proteins work methodically participating in exocytosis [10]. Several GTP binding proteins, for instance, rac, rho, and G alpha i3, participate in secretion [27, 28]. These proteins are also involved in other biological functions, so SCRN1 might promote cell proliferation and clone formation through an indirect manner.

### 3.5. Silencing of SCRN1 Expression Inhibited Cell Invasion* In Vitro*

**Figure 3:**
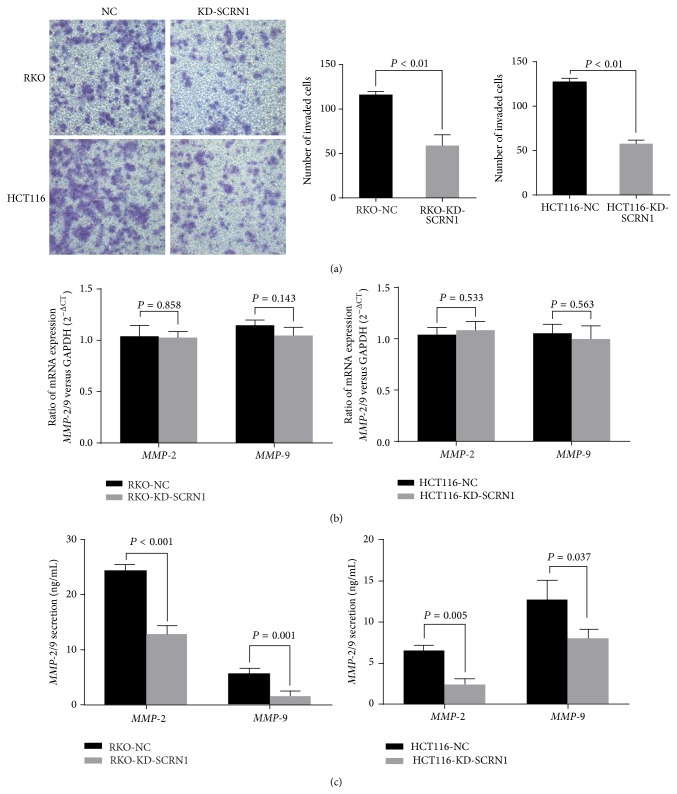
Silencing SCRN1 expression inhibited cell invasion. (a) Transwell invasion assay was performed to measure cell invasion in RKO and HCT cells. Number of invaded cells was significantly reduced due to SCRN1 silence. Data was presented as mean ± SD, *P* < 0.01 in both cases. (b) Real-time PCR revealed that no significant difference in* MMP-*2/9 mRNA expression was observed between negative control and SCNR1 knockdown cells. Data was presented as mean ± SD, *P* > 0.05 in both cases. (c) Elisa assay revealed that SCRN1 knockdown reduced* MMP*-2 protein secretion in both RKO and HCT116 cells. Results are presented as means ± SD and *P* < 0.05 in both cases.

In cell invasion assay, cells transiently transfected with SCRN1 siRNA showed a decrease in numbers of invaded cells. Silencing of SCRN1 significantly reduced the invaded cell of RKO cells and HCT116 cells by about 50% (*P* < 0.01 in both cases) (Figure 3(a)). These results indicated that SCRN1 expression is correlated with cancer cell proliferation and invasion in colon cancer.

### 3.6. Silencing of SCRN1 Has No Influence on* MMP*-2/9 mRNA Expression but Reduces* MMP*-2/9 Protein Secretion in Cells

Quantitative real-time PCR analysis was performed to determine the effect of SCRN1 on* MMP-2/9* gene expression. No decrease in* MMP-2/9* mRNA expression was observed in RKO-KD-SCRN1 and HCT116-KD-SCRN1 cells compared to RKO-NC and HCT116-NC cells (Figure 3(b)). ELISA was then conducted to confirm the total amounts of secreted* MMP*-2/9 proteins. Significant inhibition of* MMP*-2/9 secretion was observed in RKO-KD-SCRN1 and HCT-KD-SCRN1 cells compared to that of RKO-NC and HCT116-NC cells (*P* < 0.05 in both cases) (Figure 3(c)). The unusual result indicated that SCRN1 has little impact on* MMP-2/9* synthesis but enhances the exocytosis of* MMP-2/9* protein.

## 4. Discussion

SCRN1 is a member of the secretin gene family, which contains 3 genes (termed Secernin-1 through Secernin-3) localized, respectively, on chromosomes 7 (7p14.3-p14.1), 17 (17q21.3), and 2 (2p14-q14.3). SCRN1 protein has a molecular weight of 50 kDa and is expressed in the brain, prostate, thymus gland, and intestine. Previous studies demonstrated that SCRN1 is involved in the regulation of exocytosis in mast cells [8, 9]. The SCRN1 gene product has been shown to be overexpressed in gastric and colon cancer [10, 11]. However, the expression pattern and cellular localization of SCRN1 remain unclear. Additionally, only few studies addressing the significance of SCRN1 and its mechanism of action in the progression of colon cancer have been reported.

In this study, in agreement with a previous study [11], we demonstrated that SCRN1 expression is upregulated in colon cancer tissues compared to paired normal mucosa using qPCR and western blot. Using immunohistochemistry, we confirmed that SCRN1 protein was overexpressed mainly in the cell cytoplasm and that the staining intensity and extent were lower in normal mucosa than in colon cancer tissues and LNM. SCRN1 protein expression was moderate to strong in 53.0% (62/117) of colon cancer tissues.

Advanced T stage, LNM, distant metastasis, and poor differentiation are known as the main factors for poor prognosis in cancer patients. Moreover, recurrence is the major cause of therapy failure in patients with colon cancer after surgery [4, 5]. SCRN1 expression levels were reported to be correlated with poor prognosis in synovial sarcoma and colon cancer [9, 11]. In this study, we found that elevated SCRN1 expression correlated with several clinicopathological features, including T stage, N stage, distant metastasis, and AJCC stage in TMAs. These results showed that SCRN1 is closely associated with tumor progression and may demonstrate the potential of SCRN1 as a prognosis predictor in colon cancer. In contrast, previous research showed that SCRN1 expression did not present any correlation with these clinicopathological features [11]. This discrepancy may be associated with the differences between mRNA and protein levels. The differences underlying biological behaviors between colon and rectum cancer may also be responsible for this discrepancy. The use of a larger number of specimens and high-throughput screening reduced the bias in this study. Furthermore, Kaplan-Meier survival curves for OS and DFS showed that high SCRN1 expression resulted in poorer OS and DFS prognosis (*P* = 0.005 and *P* = 0.030, resp.). Elevated SCRN1 expression was associated with an increased risk of metastasis or local recurrence and was strongly linked to poor prognosis, with hazard ratios of 2.849 for OS and 2.328 for DFS in the univariate analysis. In multivariate analysis, SCRN1 expression was confirmed to be an independent prognosis predictor in colon cancer. Our results are in agreement with those of previous studies [9, 11] and the relationship between SCRN1 expression and clinicopathological features. Therefore, patients with elevated SCRN1 expression may need more powerful chemotherapy or other adjuvant therapies and intensive follow-up. Furthermore, inhibiting SCRN1 expression may be a novel targeted therapy to prevent tumor metastasis and to improve the outcomes of patients with colon cancer.

Distant metastasis and lymph node metastasis lead to poor prognosis in colon cancer [4, 5]. Early studies described that exocytosis promotes tumor metastasis and cancer cell invasion [29, 30].* MMP*-2/9 exocytosis can be regulated to alter the invasive behavior of cancer cells in breast cancer, glioma, and other tumors [20, 21, 31, 32]. Taking the SCRN1 function as a regulator of exocytosis in other cell types into consideration, we hypothesized that SCRN1 may enhance the secretion of* MMP*-2/9 to promote cancer cell invasion and tumor metastasis. SCRN1 expression was knocked down by RNA interference-mediated silencing in RKO and HCT116 cells. MTT, clone formation, and transwell invasion assays revealed that SCRN1 expression knockdown inhibited RKO cell proliferation and invasion. We then explored the effect of SCRN1 knockdown on* MMP*-2/9 expression in cells with different levels of SCRN1 expression. qPCR revealed that no significant effect was observed on mRNA expression, which demonstrated that SCRN1 has no effect on* MMP*-2/9 mRNA expression in colon cancer cells. However, our ELISA results demonstrated that* MMP*-2 and* MMP*-9 secretion were inhibited in SCRN1 silenced cells. These results demonstrate that SCRN1 alters the biological behavior of colon cancer cells through enhancing the secretion of* MMP*-2/9. However, whether SCRN1 influences the biological behavior of colon cancer cells through other mechanisms remains unclear. Future studies will be designed to properly determine the exact mechanism by which SCRN1 affects* MMP*-2/9.

In conclusion, our results demonstrated that SCRN1 mRNA and protein expression were elevated in colon cancer. In addition, we identified the significance of SCRN1 in colon cancer progression for the first time. SCNR1 overexpression serves as an independent prognostic predictor for DFS and OS in patients with colon cancer after surgery. Moreover, we reported that SCRN1 promotes colon cell proliferation and enhances the secretion of* MMP*-2/9 to accelerate cancer cell invasion and tumor metastasis.

## Figures and Tables

**Table 1:** Expression of SCRN1 protein in normal mucosa, cancerous tissue, and LNM tissues.

Tissue sample	*n*	SCRN1 expression		*P* value
		Negative and weak (*n*, %)	Moderate and strong (*n*, %)	
Normal mucosa	117	83 (70.9%)	34 (29.1%)	
Cancerous tissue	117	55 (47.0%)	62 (53.0%)	<0.001^a^
LNM tissue	42	17 (40.5%)	25 (59.5%)	<0.001^b^

SCRN1: Secernin-1; LNM: lymph node metastasis.

^a^Significant difference between cancerous tissues and normal mucosa in SCRN1 expression.

^b^Significant difference between LNM tissues and normal mucosa in SCRN1 expression.

**Table 2:** Associations of SCRN1 expression with clinicopathological features in colon cancer (*n* = 117).

Variables	*n*	SCRN1 expression		*P* value
		Negative and weak (*n* = 55)	Moderate and strong (*n* = 62)	
Age				
<65 y	50	23	27	0.850
≥65 y	67	32	35	
Gender				
Male	55	27	28	0.671
Female	62	28	34	
Location				
Right	45	20	25	
Transverse	8	4	4	0.917
Left	64	31	33	
T stage				
T1	5	2	3	0.013^*^
T2	18	10	8	
T3	45	28	17	
T4	49	15	34	
N stage				
N0	64	36	28	
N1	37	16	21	0.023^*^
N2	16	3	13	
M stage				
M0	102	52	50	0.025^*^
M1	15	3	12	
AJCC stage				
I and II	63	36	27	0.018^*^
III and IV	54	19	35	
Differentiation				
Well	60	32	28	
Moderate	38	17	21	0.240
Poorly	19	6	13	
Vessel invasion				
No	113	54	59	0.621
Yes	4	1	3	

SCRN1: Secernin-1; AJCC: American Joint Committee on Cancer.

*P* values are based on chi-square test or Fisher's exact test if necessary.

^*^means significant difference.

**Table 3:** Cox proportional hazards model univariate and multivariate analyses of individual parameters for correlations with overall survival (OS) in 117 patients.

Variable	Univariate	*P* value	Multivariate	*P* value
	HR (95% CI)		HR (95% CI)	
Age				
<65y	—		NR	
≥65y	1.243 (0.607–2.545)	0.551		
Gender				
Male	—		NR	
Female	1.625 (0.792–3.330)	0.185		
Location				
Right	—			
Transverse	0.838 (0.189–3.716)	0.816	NR	
Left	0.827 (0.402–1.703)	0.606		
T stage				
T1 and T2	—		NR	
T3 and T4	8.275 (1.129–60.629)	0.038^*^		
N stage				
N0	—			
N1	5.927 (2.109–16.654)	0.001^*^	NR	
N2	25.839 (9.067–73.633)	<0.001^*^		
M stage				
M0	—		NR	
M1	42.120 (17.057–104.009)	<0.001^*^		
AJCC stage				
I and II	—		—	
III and IV	12.242 (4.277–35.041)	<0.001^*^	8.936 (3.049–26.188)	<0.001^*^
Differentiation				
Well	—		—	
Moderate	2.045 (0.804–5.201)	0.133	1.402 (0.544–3.616)	0.484
Poorly	10.417 (4.319–25.125)	<0.001^*^	7.419 (2.971–18.524)	<0.001^*^
Vessel invasion				
No	—		—	
Yes	4.355 (1.319–14.382)	0.016^*^	1.905 (0.559–6.497)	0.303
SCRN1 expression				
Negative and weak	—		—	
Moderate and strong	2.849 (1.313–6.184)	0.008^*^	2.827 (1.228–6.507)	0.015^*^

AJCC: American Joint Committee on Cancer; SCRN1: Secernin-1;

HR: hazard ratio; CI: confidence interval. NR variable was not included in the resultant model.

*P* < 0.05 indicated that the 95% CI of HR was not including 1.

^*^means significant difference.

**Table 4:** Cox proportional hazards model univariate and multivariate analyses of individual parameters for correlations with disease-free survival (DFS) in 117 patients.

Variable	Univariate	*P* value	Multivariate	*P* value
	HR (95% CI)		HR (95% CI)	
Age				
<65 y	—		NR	
≥65 y	1.608 (0.732–3.532)	0.237		
Gender				
Male	—		NR	
Female	1.490 (0.711–3.122)	0.290		
Location				
Right	—			
Transverse	0.438 (0.057–3.365)	0.427	NR	
Left	0.831 (0.393–1.757)	0.628		
T stage				
T1 and T2	—		NR	
T3 and T4	2.437 (0.737–8.053)	0.144		
N stage				
N0	—			
N1	3.722 (1.440–9.620)	0.007^*^	NR	
N2	17.423 (6.534–46.460)	<0.001^*^		
M stage				
M0	—		NR	
M1	26.601 (10.073–70.248)	<0.001^*^		
AJCC stage				
I and II	—		—	
III and IV	5.964 (2.537–14.023)	<0.001^*^	5.041 (2.112–12.030)	<0.001^*^
Differentiation				
Well	—		—	
Moderate	2.035 (0.843–4.913)	0.114	1.589 (0.653–3.864)	0.307
Poorly	6.404 (2.521–16.268)	<0.001^*^	5.635 (2.143–14.821)	<0.001^*^
Vessel invasion				
No	—		NR	
Yes	3.954 (0.934–16.738)	0.062		
SCRN1 expression				
Negative and weak	—		—	
Moderate and strong	2.328 (1.059–5.114)	<0.035^*^	2.262 (1.008–5.078)	<0.048^*^

AJCC: American Joint Committee on Cancer; SCRN1: Secernin-1;

HR: hazard ratio; CI: confidence interval. NR variable was not included in the resultant model.

*P* < 0.05 indicated that the 95% CI of HR was not including 1.

^*^means significant difference.
